# Variation in species‐level plant functional traits over wetland indicator status categories

**DOI:** 10.1002/ece3.2975

**Published:** 2017-04-17

**Authors:** Miles E. McCoy‐Sulentic, Thomas E. Kolb, David M. Merritt, Emily C. Palmquist, Barbara E. Ralston, Daniel A. Sarr

**Affiliations:** ^1^ School of ForestryNorthern Arizona UniversityFort CollinsUSA; ^2^ USDA Forest ServiceNational Stream and Aquatic Ecology CenterFort CollinsCOUSA; ^3^ US Geological SurveySouthwest Biological Science CenterFlagstaffAZUSA; ^4^ US Geological SurveyOffice of Science Quality and IntegrityFlagstaffAZUSA

**Keywords:** Arizona, Grand Canyon, inundation, plant trait, riparian, water use efficiency

## Abstract

Wetland indicator status (WIS) describes the habitat affinity of plant species and is used in wetland delineations and resource inventories. Understanding how species‐level functional traits vary across WIS categories may improve designations, elucidate mechanisms of adaptation, and explain habitat optima and niche. We investigated differences in species‐level traits of riparian flora across WIS categories, extending their application to indicate hydrologic habitat. We measured or compiled data on specific leaf area (SLA), stem specific gravity (SSG), seed mass, and mature height of 110 plant species that occur along the Colorado River in Grand Canyon, Arizona. Additionally, we measured leaf δ^13^C, δ^15^N, % carbon, % nitrogen, and C/N ratio of 56 species with C3 photosynthesis. We asked the following: (i) How do species‐level traits vary over WIS categories? (ii) Does the pattern differ between herbaceous and woody species? (iii) How well do multivariate traits define WIS categories? (iv) Which traits are correlated? The largest trait differences among WIS categories for herbaceous species occurred for SSG, seed mass, % leaf carbon and height, and for woody species occurred for height, SSG, and δ^13^C. SSG increased and height decreased with habitat aridity for both woody and herbaceous species. The δ^13^C and hence water use efficiency of woody species increased with habitat aridity. Water use efficiency of herbaceous species increased with habitat aridity via greater occurrence of C4 grasses. Multivariate trait assemblages differed among WIS categories. Over all species, SLA was correlated with height, δ^13^C, % leaf N, and C/N; height was correlated with SSG and % leaf C; SSG was correlated with % leaf C. Adaptations of both herbaceous and woody riparian species to wet, frequently inundated habitats include low‐density stem tissue. Adaptations to drier habitats in the riparian zone include short, high‐density cavitation‐resistant stem tissue, and high water use efficiency. The results enhance understanding about using traits to describe plant habitat in riparian systems.

## 1 Introduction

The use of plant functional traits in ecology aims to elucidate links between species’ characteristics, habitat, distribution, and community assembly (Grime, 1977; Poff, 1997; Ackerly, Knight, Weiss, Barton, & Starmer, 2002; Merritt, Scott, Poff, Auble, & Lytle, 2010). This use is founded on the theory that taxonomically unrelated species show convergent adaptations to similar environmental conditions and assumes that the distributions of plant traits over environmental gradients are predictable and explain the mechanisms defining the niche of a species (Keddy, 1992; Reich, Walters, & Ellsworth, 1997; Reich et al., 2003; Shipley et al., 2016). A growing number of studies report consistent trends in interspecific variation in traits across environmental gradients at regional to global scales (Chave et al., 2009; Kattge et al., 2011; Wright et al., 2004) and have provided support to plant strategy theory by elucidating trade‐offs in traits for establishment and persistence in different environments (Grime, 1977; Lavorel & Garnier, 2002; Westoby & Wright, 2006). However, the extent to which relationships between functional traits and environmental gradients apply to specific regional and local contexts warrants further exploration (Shipley et al., 2016).

Riparian ecosystems in arid regions are ideal for investigating species‐level trait variation across habitats to understand adaptation to inundation, flooding, and aridity. These ecosystems are highly species diverse relative to upland plant communities and include sharp spatial gradients of inundation, fluvial disturbance, and water availability across relatively short distances within a river valley (Horton, Kolb, & Hart, 2001; Naiman, Decamps, & Pollock, 1993; Shafroth, Stromberg, & Patten, 2002). Because hydrologic and geomorphic characteristics define the dominant disturbance regime of riparian ecosystems (i.e., flooding), vegetation communities adjacent to rivers are often arranged in elevational bands along gradients of disturbance and moisture availability (Nilsson & Svedmark, 2002; Sluis & Tandarich, 2004). Riparian plants in these communities display a range of morphological and physiological adaptations that influence their growth, reproduction, water balance, and survival in conditions ranging from drought to inundation and prolonged submergence (Catford & Jansson, 2014; Lytle & Poff, 2004).

In the United States, a national wetland plant list was established in 1988 as part of a wetland classification system widely used in resource inventories, wetland delineations, and research (Cowardin, Carter, Golet, & LaRoe, 1979; Reed, 1988). The plant list is periodically updated and is publically available (Lichvar, Butterwick, Kirchner, & Melvin, 2016) and consists of categorical designations of the most typical hydrologic habitat in wetland and riparian areas for hundreds of plant species based on expert opinions. These designations are often used to identify wetland habitat. Following Stromberg (2013), we extend the use of these designations to describe the hydrologic habitat in which each species is likely to occur. Investigation of quantitative functional traits of species on the plant list may elucidate the underpinnings of designations, facilitate classification of new species, and refine the overall designation scheme.

Our study aims to understand mechanisms of adaptation by comparing traits of species across wetland indicator status (WIS) categories (Lichvar et al., 2016; Reed, 1988). For example, Stromberg (2013) used species‐level comparisons among WIS categories to show that obligate riparian species have shallower roots than facultative (FAC) riparian species and thus demonstrated a functional linkage between a plant morphological characteristic and niche. A small number of measurable traits have been postulated to represent the myriad of plant functional strategies and trait syndromes (Weiher et al., 1999; Westoby, 1998). For example, the leaf–height–seed scheme uses specific leaf area (SLA), plant maximum height, and seed mass to condense variation in a wider suite of traits and quantify plant strategy (Westoby, 1998). SLA represents the amount of leaf area per investment in leaf mass and is correlated with photosynthetic capacity, nitrogen content, leaf longevity, and leaf architecture (Ordoñez et al., 2009; Westoby, 1998). Low SLA has been reported in habitats that are arid (Wright et al., 2004), infertile (Hodgson et al., 2011), open (Ackerly, Knight, Weiss, Barton, & Starmer, 2002), and in more drought tolerant species (Mitchell, Veneklaas, Lambers, & Burgess, 2008). High SLA, in contrast, has been associated with wetter habitats (Cornwell & Ackerly, 2009) and may promote leaf gas exchange in plants coping with submergence (Mommer, Wolters‐Arts, Andersen, Visser, & Pedersen, 2007; Voesenek, Colmer, Pierik, Millenaar, & Peeters, 2006). Plant height is related to competitive ability for light and soil resources through the expectation of larger root systems for taller plants (Keddy & Shipley, 1989; Westoby, 1998). Tall height also may be an adaptation to flooding by allowing less complete and shorter duration submergence of the stem (Voesenek et al., 2006). Seed mass is related to reproductive strategy, drought tolerance, and seedling establishment (Larios, Búrquez, Becerra, & Lawrence Venable, 2014; Leishman, Westoby, & Jurado, 1995; Westoby, 1998). Larger seed size may confer greater drought tolerance and survivorship by providing greater metabolic reserves for root elongation and construction of thick cell walls during seedling establishment and often occurs in species that occupy less disturbed sites and in areas where competition is more intense (Leishman & Westoby, 1994; Westoby, Jurado, & Leishman, 1992).

Other traits associated with water use and drought tolerance may also be informative in understanding adaptations of riparian plants. Wood density, or stem specific gravity (SSG), is correlated with xylem cell characteristics (Hacke, Sperry, Pockman, Davis, & McCulloh, 2001), leaf size (Pickup, Westoby, & Basden, 2005), growth rate (Poorter et al., 2010), soil water availability (Preston, Cornwell, & DeNoyer, 2006), and cavitation resistance in both woody and herbaceous species (Hacke et al., 2001; Jacobsen, Esler, Pratt, & Ewers, 2009; Lens et al., 2016; Pockman & Sperry, 2000; Wahl & Ryer, 2000). Carbon isotope ratio of leaf tissue is a time‐integrated measure of leaf internal CO_2_ concentration and stomatal regulation in C3 plants and is correlated with water use efficiency (Cernusak et al., 2013; Diefendorf, Mueller, Wing, Koch, & Freeman, 2010; Martínez‐Vilalta, Sala, & Piñol, 2004). Leaf δ^15^N can provide information about inundation frequency and soil mineralization rates (Austin et al., 2004; Hall, Hale, Baker, Bowling, & Ehleringer, 2015). Foliar carbon and nitrogen concentrations reflect structural and photosynthetic investments in leaves and can be correlated with photosynthetic rate, leaf longevity, and decomposition rate (Pérez‐Harguindeguy et al., 2013; Reich, 2014).

Our study focuses on riparian vegetation of the Colorado River in Grand Canyon in northern Arizona where completion of Glen Canyon Dam in 1964 altered flood frequency and magnitude as well as sediment regime and transport processes, severely altering the historic disturbance regime and leading to vegetation establishment, encroachment and narrowing of the active river channel (Gloss, Lovich, & Melis, 2005; Sankey, Ralston, Grams, Schmidt, & Cagney, 2015; Topping, Schmidt, & Vierra, 2003; Turner & Karpiscak, 1980). Additionally, river regulation created a permanent base flow that enabled the establishment and proliferation of plant species that were historically absent or rare (Stevens, Schmidt, Ayers, & Brown, 1995). Predictions of a warmer future climate and changes in water availability (e.g., reduced headwater snowpack and timing of flows) in this arid region may cause further alterations in river flows and the riparian vegetation community (Dominguez, Rivera, Lettenmaier, & Castro, 2012; Hayhoe et al., 2004; Seager & Vecchi, 2010; Seager et al., 2007). Better knowledge about distributions of species’ functional traits across gradients of water availability and inundation is needed in order to inform predictions of plant community changes in response to further alterations to river flows and changing moisture availability and temperature and may help refine wetland plant classification.

We compared nine traits (SSG, seed mass, plant height, SLA, foliar δ^13^C and δ^15^N, foliar carbon and nitrogen concentration, foliar carbon‐to‐nitrogen ratio) of 110 plant species that commonly occur along the Colorado River in Grand Canyon National Park, Arizona among five WIS categories (Lichvar et al., 2016; Reed, 1988). These categories describe the most typical habitat of a plant species and the affinity of species for conditions ranging between frequently flooded, inundated areas to arid habitats in uplands adjacent to the river. The species we investigated include a mixture of natives and non‐natives, many of which occur in riparian ecosystems throughout the world. Thus, our study, while focused on riparian flora at a specific river, has broader application for understanding adaptations of riparian plants to environmental gradients. We addressed four questions: (i) How do species‐level functional traits related to dispersal strategy, competitive ability, leaf physiology, water relations, and drought tolerance of Colorado River riparian flora vary over WIS categories? (ii) Does the pattern differ between herbaceous and woody species? (iii) How well do multivariate traits define WIS categories? (iv) Which traits are correlated?

## 2 Materials and Methods

### 2.1 Site description

Our study area is 362 km of the Colorado River between Lees Ferry and Diamond Creek, AZ. This includes all of Marble Canyon and much of Grand Canyon (referred to collectively as Grand Canyon). Vegetation communities vary along the river and are comprised of species found in the Sonoran, Great Basin, and Mojave Deserts (Huisinga, Makarick, & Watters, 2006; McLaughlin, 1986). The frequency and magnitude of flooding has been greatly reduced since the construction of Glen Canyon Dam, and variation in discharge is largely controlled by dam operations (Gloss et al., 2005). The river channel averages 100 meters wide and is largely confined by bedrock (Howard & Dolan, 1981). There is a steep moisture gradient from perpetually flooded areas in the active channel and high desert environments on colluvial side slopes and predam fluvial deposits furthest from the channel. Channel constrictions occurring at side canyons (debris fans) resulting from debris flows reduce the river's velocity and allow for deposition of sediments and formation of sandbars downstream from fans (Dolan, Howard, & Gallenson, 1974; Howard & Dolan, 1981; Schmidt, 1990). These sandbars form habitats within the channel and, in conjunction with reductions in flooding and increases and consistency of baseflows, have facilitated the establishment of marshes and other vegetation typical of wetland habitats along the channel and the encroachment of vegetation onto previously active, unvegetated sandbars (Gloss et al., 2005; Sankey et al., 2015; Stevens et al., 1995; Turner & Karpiscak, 1980).

### 2.2 Data collection

We used riparian vegetation surveys conducted by the US Geological Survey (USGS) at sandbars along the Colorado River within the Grand Canyon National Park to guide species selection. We measured or compiled data for 110 species (see Table 1 for summary of traits, see Appendices S1 and S2 for a list of species sampled and sampling locations), which included those that were most commonly documented in vegetation monitoring surveys in years 2012–2014, as well as many less common species. The species include six trees with the C3 photosynthetic pathway (C3), 18 C3 woody shrubs, 16 C3 grasses, 38 C3 forbs, three C3 herbaceous subshrubs, one woody shrub with the C4 photosynthetic pathway (C4), 22 C4 grasses, two C4 forbs, and three forbs and one subshrub of undetermined photosynthetic pathway.

**Table 1:** Traits used for this study, their units, source, and brief interpretation

Trait	Unit	Source	Interpretation
Seed mass	grams per 1,000 seeds	Kew database	Reproductive strategy, drought tolerance
Specific leaf area	cm^2^/g^1^	Field samples	Leaf economic strategy, gas exchange
Stem specific gravity	g/cm^3^	Field samples	Drought tolerance
Plant height	m	FNA, Jepson, Utah Flora	Competitive ability
δ^13^C	‰	Field samples	Water use efficiency
δ^15^N	‰	Field samples	Nitrogen source
% Carbon	% mass of sample	Field samples	Structure investment, decomposition rate
% Nitrogen	% mass of sample	Field samples	Photosynthesis
Carbon/nitrogen ratio	g/g	Field samples	Structural investment, decomposition

Trait data for the project are available at, https://doi.org/10.5066/F7BV7DTQ.

Our field sampling approach emphasized estimation of mean trait values at the species level and did not formally consider intraspecific variation (e.g., among habitats for a given species) based on recent findings that trait rankings of species in large datasets typically are not sensitive to the exact location of sampling (Kazakou et al., 2014; Ordoñez, 2014). We sampled most (81%, or 89 species) species along the Colorado River, whereas 19% (21 species) were sampled along tributaries or at other locations in northern Arizona (Appendix S1 and S2). We sampled five individuals from one representative population of each species to characterize mean trait values. We maximized the trade‐off between number of species, time for sample preparation and analysis, and number of individuals per species, concluding that five samples per species would well‐represent the dominant species. Representative populations were defined as temporally persistent populations of apparently vigorous plants based on our many years of plant surveys at the study sites. We targeted individuals that were mature, had healthy appearance, and were in locations typical for the species (Pérez‐Harguindeguy et al., 2013). We did not sample species located in suboptimal habitat or with visible signs of water stress, disease, or insect or herbivore damage. While this sampling approach has limitations in not capturing intraspecific variation in trait values potentially caused by environmental variation among sites and intraspecific genetic variation, it facilitated our inclusion of a large number of plant species (110) that grow in a rugged landscape where complete sampling of environmental gradients is extremely difficult. Plant tissue collected in the field was kept in sealed plastic bags with a damp paper towel, inside coolers with ice during collection trips and transportation back to the laboratory. Once returned, samples were kept cool at 2–6°C until measurements were made.

We measured SLA on four fully developed leaves from each of five different individuals, and the individual averages were used to calculate a species average. SLA was calculated as the projected leaf area per dry weight of biomass (cm^2^/g). Leaves were scanned (petioles included) using a flatbed scanner and Winfolia software (Version 2007b, Regent Instruments, Inc.) to determine leaf area and then oven‐dried at 70°C for 72 hr and weighed to determine dry mass. Exceptions to this protocol included species of *Tamarix*,* Ephedra*,* Equisetum*, and *Juncus*. For *Tamarix* spp., which have a decussate leaf arrangement with very small leaves, a heavily foliated branchlet located near the end of a branch was used rather than an individual leaf scale. For species of *Ephedra*,* Equisteum*, and *Juncus*, which have photosynthetic stems or vertical linear leaves, a photosynthetic stem segment (variable length depending on species) was used.

Whole‐stem cross sections from the base of the stem of both woody shrubs and herbaceous species, generally about 5 cm in length, were used for measurement of SSG. Exceptions to this protocol included species of *Juncus* and *Plantago*, for which the base of the leaf was treated as the stem. We used average SSG values for individual trees using measurements from sapwood obtained from increment cores and one branch cross section. For the six tree species sampled (*Acacia greggii, Prosopis glandulosa, Populus fremontii, Celtis laevigata, Tamarix* spp.*, and Salix gooddingii*), only *C. laevigata* had a significant difference in SSG between cores and branch cross sections (*t* = 4.149, *p *= .003). Measurement of SSG first required measurement of stem volume on a fresh sample using an analytical balance and the water displacement method (Pérez‐Harguindeguy et al., 2013). A small segment was submerged by hand with a small pin and the change in mass recorded. Sample volume was recorded as the displaced mass of the water based on the density of water (Williamson & Wiemann, 2010). Herbaceous stems were dried at 70°C for 72 hr (Pérez‐Harguindeguy et al., 2013) and woody stems were dried at 103°C for 72 hr (Williamson & Wiemann, 2010) before measurement of mass. The SSG was calculated from these measurements as dry mass per volume (g/cm^3^).

We obtained data on height and seed mass of the species from previously compiled databases and flora. Plant heights were compiled from floral descriptions found in A Utah Flora (Welsh, Atwood, Goodrich, & Higgins, 1993), the Jepson Desert Manual (Baldwin, 2002), and the Flora of North America (Flora of North America Editorial Committee 1993). Height was calculated as the average of the maximum and minimum height reported for mature plants in these floras. We found height data from this source to be strongly correlated with heights, we measured directly on species at field collection sites (Spearman *r*_s_ = .83, *p *<* *.05). Seed mass data were compiled from the KEW Royal Botanic Garden Seed Information Database (http://data.kew.org/sid/) and consist of the average weight in grams per 1,000 seeds.

We measured additional foliar traits (δ^13^C, δ^15^N, percent carbon and nitrogen, and carbon/nitrogen ratio) on 56 species that have the C3 photosynthetic pathway. These additional measurements focused on species with the C3 photosynthetic pathway because differences in δ^13^C can be more directly interpreted as changes in leaf gas exchange and water use efficiency in C3 species than in C4 species (Cernusak et al., 2013). Leaves used in measurement of SLA were also used for isotope analysis after measurement of SLA. For species with small leaves, extra leaves of healthy appearance were sampled from the same individuals and used to meet material quantity requirements for isotopic analysis. A minimum of three individuals (typically five) per species were used for isotope analysis and to estimate average trait values for the species. Leaf samples were analyzed using a Thermo‐Electron Delta V Advantage isotope ratio mass spectrometer by the Colorado Plateau Stable Isotope Laboratory (CPSIL) at Northern Arizona University. Leaf carbon and nitrogen are expressed as percent of sample dry weight, and δ^13^C and δ^15^N are expressed as parts per mil relative to International Atomic Energy Agency standards of Vienna Pee Dee Belemnite (VPDB) and atmospheric N_2_, respectively.

### 2.3 Wetland indicator status

We used existing WIS categories to classify the typical habitat of each species. We used this approach because WIS is available for many species, it is based on many expert opinions, and it is often used in regulation. The list of species in each category was developed by the US Army Corps of Engineers (USACE), US Fish and Wildlife Service, US Environmental Protection Agency, and Natural Resources Conservation Service using expert opinion and taxonomic and distribution data from the Biota of North America Program (http://www.bonap.org/). The list is published by the USACE as part of the National Wetland Plant List (Lichvar et al., 2016; Reed, 1988) and is available from websites maintained by the USACE (http://wetland_plants.usace.army.mil/) and the US Department of Agriculture PLANTS database (www.plants.usda.gov). The categories are obligate wetland (OBL)—almost always occur in wetlands, facultative wetland (FACW)—usually occur in wetlands, but may occur outside of wetlands, FAC—occur in wetlands and nonwetlands, facultative upland (FACU)—usually occur in nonwetlands, but may occur in wetlands, and UPL (obligate upland)—almost never occur in wetlands. OBL and FACW species are considered hydrophytes, FAC species can occur in both wetlands and nonwetlands, whereas FACU and UPL species are considered nonhydrophytes (USDA 2017). Although these categories are widely used in the United States to describe typical habitats of species, their use as a proxy of habitat may have limitations because of inaccuracy in classifying some species, and because they may not adequately represent all aspects of habitat, such as disturbances.

### 2.4 Data analysis

We compared species‐level traits over WIS categories. Comparisons were carried out for all species pooled and for woody and herbaceous species separately. We tested for differences in traits among categories using ANOVA and Tukey's Honestly Significant Difference (HSD) for data that met normality and variance assumptions, and nonparametric Kruskal–Wallis and Wilcoxon rank tests for data that were not normally distributed. Data were analyzed, and results are presented in original units of measurement, except for seed mass in which ranks were used for analysis and presentation to facilitate visual presentation due to extreme variation among species in absolute values. Spearman rank correlations (*r*_s_) were used to examine relationships among traits. These statistical tests were performed using R software (Version 0.98.1103, R Core Team, 2013) and the car (Fox & Weisberg 2011) and Hmisc (Harrell 2015) packages.

To test how multivariate trait assemblages differed over WIS categories, we used permutation multivariate analysis of variance (PERMANOVA, PRIMER software v7, Anderson, 2001; Clarke & Gorley, 2015). A reduced number of traits consisting of SLA, height, seed mass, and SSG grouped by WIS were used. This smaller set of traits was used because data were available for all species and allowed greater sample size, they were strongly correlated with other traits in previous studies (Westoby, 1998), and due to their importance in plant drought tolerance (Brodribb, 2009; Hacke et al., 2001). Seed mass and height data were natural log‐transformed to improve skewed distributions, and data for all traits were standardized so that values ranged between −1 and 1. The analysis was run for 999 permutations using Euclidian distance. Principal components analysis ordination with a biplot overlay of traits was used to display the results.

## 3 Results

### 3.1 All species

**Figure 1:**
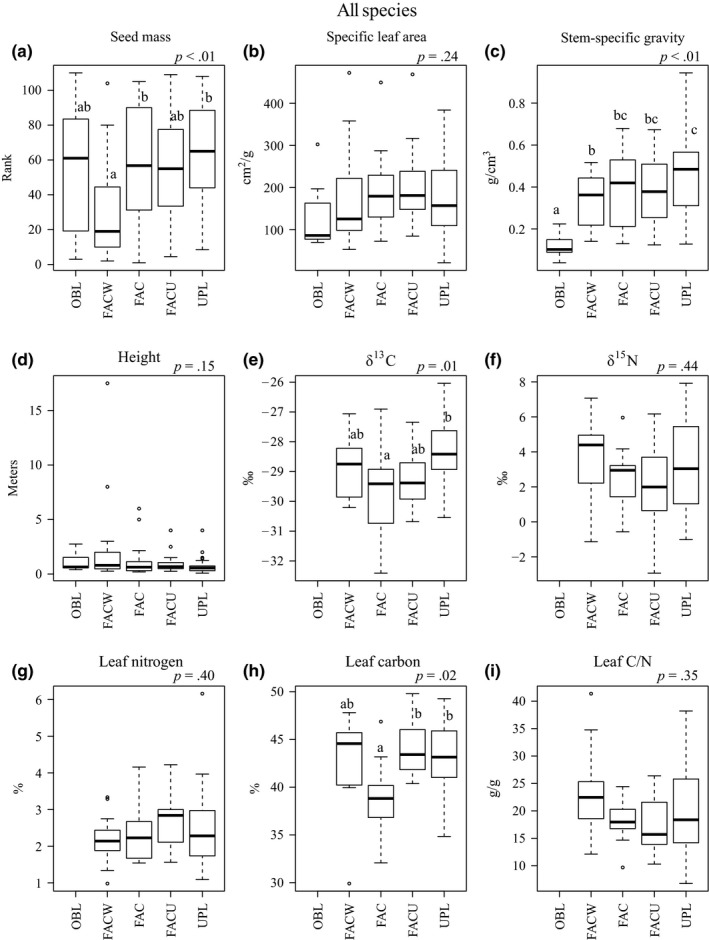
Boxplot comparisons for all species trait distributions by wetland indicator status category: OBL, Obligate; FACW, Facultative Wetland; FAC, Facultative; FACU, Facultative Upland; UPL, Upland. The *p*‐value of ANOVA or Kruskal–Wallis test reported at top right of each panel. Different letters denote significant differences based on pairwise comparisons at *p *< .05. If no letters are present, overall differences were not significant (*p *> .05). Analyses of δ^13^C, δ^15^N, % leaf nitrogen, % leaf carbon, and leaf C/N were performed for C3 species only. Sample sizes are shown in Appendix S3

For data pooled over all species, statistically significant differences in trait values among WIS categories occurred for seed mass (*x* ^2^ = 17.07, *p* < .01), SSG (*F *= 7.65, *p *< .01), δ^13^C (*F *= 3.87, *p *= .01), and % leaf carbon (*x* ^2^ = 9.82, *p *= .02; Figure 1). Seed mass was lowest in the FACW category, but similar in other categories. SSG was lowest in the OBL category, intermediate in the FACW, FAC, and FACU categories, and highest in the UPL category. The δ^13^C was lowest (most negative) in the FAC category and highest in the UPL category (OBL not included due to low sample size). Percent leaf carbon was lowest in the FAC category, but similar in other categories. While SLA tended to increase from the OBL category to the more upland categories, differences were not significant (*F *= 1.40, *p *= .24). Height, δ^15^N, % leaf nitrogen, and leaf C/N did not differ significantly among habitat categories and had no obvious trends (Figure 1).

### 3.2 Herbaceous species

**Figure 2:**
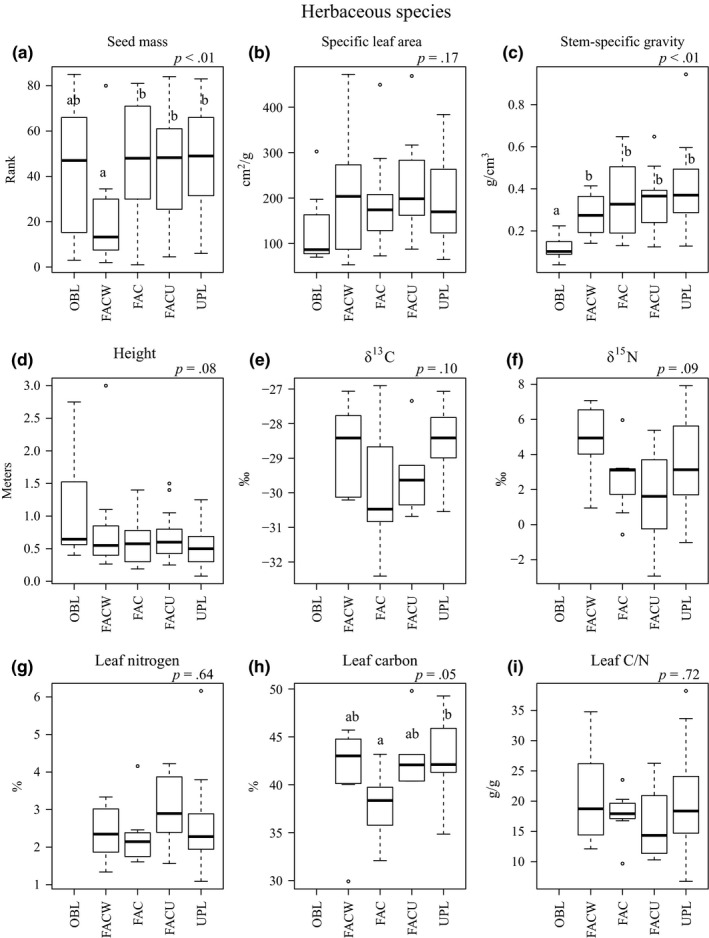
Boxplot comparisons of herbaceous species trait distributions by wetland indicator category: OBL, Obligate; FACW, Facultative Wetland; FAC, Facultative; FACU, Facultative Upland; UPL, Upland. The *p*‐value of ANOVA or Kruskal–Wallis test reported at top right of each panel. Different letters denote significant differences based on pairwise comparisons at *p *< .05. If no letters are present, overall differences were not significant (*p *> .05). Analyses of δ^13^C, δ^15^N, % leaf nitrogen, % leaf carbon, and leaf C/N were only performed for C3 species. Sample sizes are shown in Appendix S3

For herbaceous species, statistically significant differences in trait values among WIS categories occurred for seed mass (*x* ^2^ = 15.12 *p* < .01) and SSG (*x* ^2^ = 20.73, *p *< .01), with marginally significant differences and trends for height (*F *= 2.20, *p *= .08), δ^13^C (*F *= 2.27, *p *= .10), δ^15^N (*F *= 2.32 *p *= .09), and % leaf carbon (*F *= 2.88, *p *= .05; Figure 2). Seed mass was lowest in the FACW category, but similar in other categories. SSG was lowest in the OBL category and increased in more upland categories. Height generally decreased from the OBL category to more upland categories. The δ^13^C was lowest in the FAC category and highest in the UPL category. The δ^15^N was lowest in the FACU category and highest in the FACW category. Percent leaf carbon was lowest in the FAC category, but similar in other categories. SLA was lowest in the OBL category, but overall differences in SLA were not significant (*F *= 1.65, *p *= .17). Percent leaf nitrogen and leaf C/N did not differ significantly among WIS categories and had no obvious trends (Figure 2).

### 3.3 Woody species

**Figure 3:**
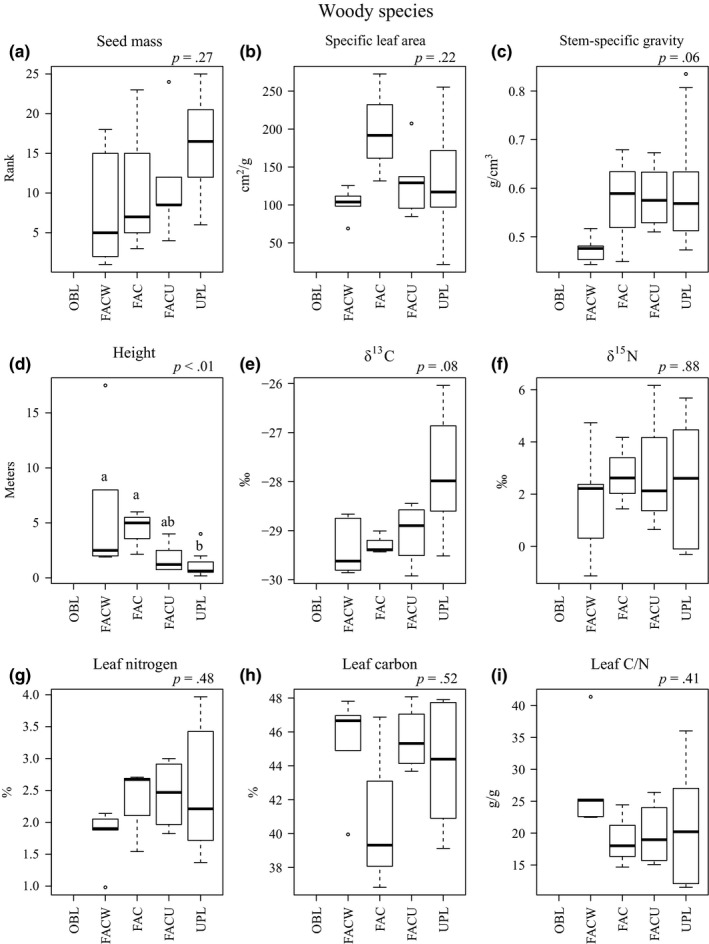
Boxplot comparisons of woody species trait distributions by wetland habitat category: OBL, Obligate; FACW, Facultative Wetland; FAC, Facultative; FACU, Facultative Upland; UPL, Upland. The *p*‐value of ANOVA or Kruskal–Wallis test reported at top right of each panel. Different letters denote significant differences based on pairwise comparisons at *p *< .05. If no letters are present, overall differences were not significant (*p *> .05). Analyses of δ^13^C, δ^15^N, % leaf nitrogen, % leaf carbon, and leaf C/N were only performed for C3 species. Sample sizes are shown in Appendix S3

For woody species, statistically significant differences in trait values among WIS categories occurred for height (*F *= 6.61, *p *< .01), with marginally significant differences and trends for SSG (*x* ^2^ = 7.47, *p *=* *.06) and δ^13^C (*x* ^2^ = 6.81, *p *=* *.08; Figure 3). SSG was lowest in the FACW category, but similar in other categories. Height was lowest in the UPL category. The δ^13^C was lowest in the FACW category and increased in more upland categories. Seed mass, SLA, δ^15^N, % leaf nitrogen, % leaf carbon, and leaf C/N did not differ significantly among WIS categories and had no obvious trends (Figure 3).

### 3.4 Multivariate analysis

**Figure 4:**
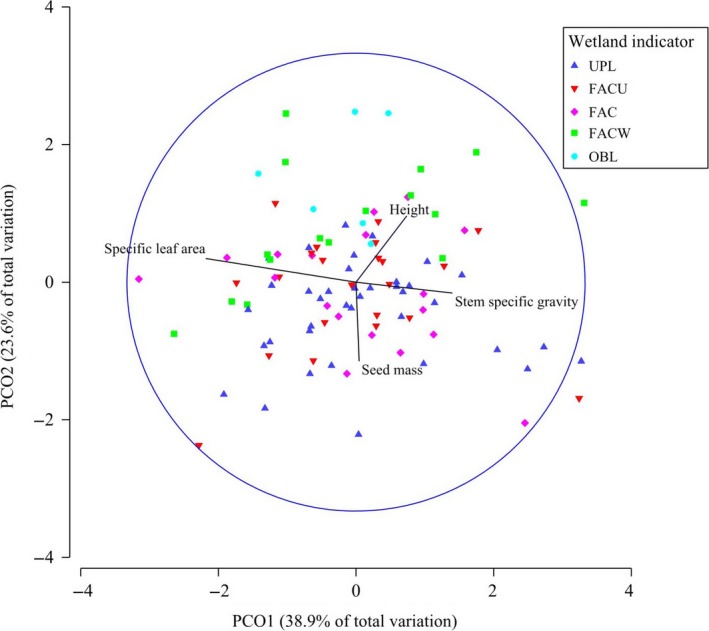
Principal components analysis of five wetland indicator status (WIS) categories using traits of height, seed mass, SLA, and SSG. PCO1, Principal component 1; PCO2, Principal component 2. Blue biplot vector lines indicate strength correlations of traits to axes. WIS categories are OBL, Obligate wetland; FACW, Facultative wetland; FAC, Facultative; FACU, Facultative upland; UPL, Upland. Sample sizes are shown in Appendix S3

The principal components analysis for data pooled over all species based on traits of height, seed mass, SLA, and SSG showed moderate separation of some WIS categories (Figure 4). PERMANOVA showed significant differences in trait suites among WIS categories (Pseudo *F *=* *3.33, *p *<* *.01), with significant pairwise differences (*p *<* *.05) distinguishing both the OBL and FACW categories from the FAC, FACU, and UPL categories. The strongest Spearman rank correlation with principal component 1 (PCO1), which explained 39% of variation in the data, was SLA (*r* = −.66), followed by SSG (*r* = .42), height (*r* = .22), and seed mass (*r* = .01). The strongest Spearman rank correlation with principal component 2 (PCO2), which explained 24% of variation, was seed mass (*r* = −.35), followed by height (*r* = .29), SLA (*r* = .10), and SSG (*r* = −0.05).

## 4 Correlation among Traits

Significant rank correlations were detected between SLA and height, δ^13^C, % leaf nitrogen, and C/N ratio (Table 2). Height was significantly correlated with SSG and % leaf carbon. SSG was significantly correlated with % leaf carbon.

**Table 2:** Spearman rank correlations between trait values for pooled species

	SLA	Height	Seed mass	SSG	δ^13^C	δ^15^N	% N	% C
Height	−0.28**							
Seed mass	−0.17	−0.004						
SSG	−0.14	0.20*	0.11					
δ^13^C	−0.3*	−0.08	0.14	0.23				
δ^15^N	0.05	−0.1	−0.22	−0.2	0.13			
% N	0.43**	−0.03	0.13	−0.06	0.17	−0.009		
% C	−0.25	0.34*	−0.19	0.51**	0.14	−0.09	−0.02	
C/N	−0.5**	0.14	−0.17	0.2	−0.07	0.03	−0.93**	0.30*

Significance level indicated by *p ≤ .05, **p ≤ .01.

## 5 Discussion

### 5.1 Trait variation among wetland indicator status categories

Our results for several functional traits of both woody and herbaceous species suggest convergent adaptation to extremely wet (OBL) and dry (upland) habitats. Herbaceous and woody species had qualitatively similar differences in SSG over WIS categories. The increase in SSG from wetland habitats to more arid upland habitats likely reflects greater cavitation resistance of plants with denser stem tissue density. This interpretation about SSG for riparian plants of Grand Canyon is consistent with other investigations of woody plants across gradients of water availability (Chave et al., 2009; Hacke et al., 2001; Kyle & Leishman, 2009; Pockman & Sperry, 2000), yet adds new information for riparian herbaceous plants of the southwestern USA. Our finding of an increase in SSG of herbaceous species from wetland habitats to drier more upland habitats is consistent with previous reports that grass species with more lignified roots (Wahl & Ryer, 2000) and stems (Lens et al., 2016) are more resistant to drought‐induced xylem cavitation and supports the hypothesis of similar xylem structure–function relationships in herbaceous and woody species (Lens et al., 2016; Tixier et al., 2013).

Our results suggest an adaptive role of seed mass to inundation and aridity in riparian ecosystems. Both herbaceous and woody species had the lowest seed mass in the FACW habitat category and greater seed mass in more arid, upland habitat categories. Seed mass of herbaceous species from the OBL category was higher than expected and similar to mass in more upland categories. Lower seed mass may be related to buoyancy and dispersal ability in plants, imparting a selective dispersal advantage for those plants growing near water (Merritt & Wohl, 2002). This finding shows that some herbaceous species with large seeds can successfully occupy the wettest sites along rivers, likely because they also have other traits that facilitate tolerance of inundation and saturated soil, such as low‐density aerenchymous stems, leaves, and roots. Overall, our results suggest that species along the Colorado River in Grand Canyon occupy more arid, less inundated sites in part by having large seeds that promote large seedlings and deep roots.

Plant height is another important functional trait for adaptation to inundation and aridity in our study. Mature plant height changed over WIS categories in a consistent pattern for herbaceous and woody species. Species’ heights decreased from the wettest to the driest habitats. For example, the facultative wetland categories (FACW, FAC) of woody plants include relatively tall species at maturity, such as *P. fremontii*,* Baccaris salicifolia*, and *Tamarix* spp., whereas the upland category (UPL) includes short desert shrubs such as *Encelia farinosa* and *Atriplex canescens*. Decreasing height with increasing habitat aridity is consistent with other reports for riparian ecosystems (e.g., Kyle & Leishman, 2009) and may be explained by greater constraints on canopy growth by water stress in upland sites, and greater below‐ground growth allocation of xerophytic vegetation (Canadell et al., 1996; Stromberg, 2013).

Results for leaf tissue δ^13^C of plants with the C3 photosynthetic pathway differed between herbaceous and woody plants. Differences in δ^13^C of herbaceous species having C3 photosynthesis were not consistently related to aridity of WIS categories, with the highest values occurring in both wet (FACW) and dry (UPL) categories. However, the proportion of herbaceous species with the C4 photosynthetic pathway increased from wet (0.0 OBL, 0.17 FACW) to dry WIS categories (0.33 FACU, 0.4 UPL). This pattern is consistent with an increase in water use efficiency of herbaceous species with habitat aridity in our study due to greater occurrence of C4 grasses in drier habitats. C4 grasses are well‐known to have higher δ^13^C and water use efficiency (Cernusak et al., 2013) and typically occupy hotter sites than C3 grasses (Gurevitch, Scheiner, & Fox, 2006). Given this interpretation of our results for herbaceous species, results for water use efficiency of woody species were similar to herbaceous species. Leaf tissue δ^13^C and hence water use efficiency of woody species consistently increased with WIS category aridity. This finding for riparian vegetation within the Grand Canyon is consistent with earlier investigations of desert riparian vegetation in southwestern North America (Ehleringer & Cooper, 1988) and comparisons among woody species over wide habitat and climatic gradients (Diefendorf et al., 2010). Overall, we conclude that both woody and herbaceous species of riparian vegetation of Grand Canyon increase water use efficiency with habitat aridity through species replacement, with this change for herbaceous species primarily driven by a shift from species with C3 photosynthesis to C4 photosynthesis.

Given the prominent role of SLA in plant strategy schemes (Grime, 1977; Westoby, 1998; Westoby & Wright, 2006), we expected large differences in SLA among WIS categories but found nonsignificant differences. While not statistically significant, SLA of both herbaceous and woody species was lowest for species most likely to be found in the wettest WIS category (OBL). Whereas higher SLA in mesic areas may be expected (Poorter, Niinemets, Poorter, Wright, & Villar, 2009; Wright et al., 2004), hydrophytic wetland species have a broad range of growth forms and SLA (Pierce, Brusa, Sartori, & Cerabolini, 2012). For example, SLA of herbaceous species in the OBL category with obvious aerenchyma tissue based on visual observations of cut stems (genera *Scirpus*,* Juncus*, and *Typha*) averaged 106 cm^2^/g and ranged from 53 to 197 cm^2^/g, whereas average SLA for the only species in the OBL category that did not have a vertical linear leaf with pronounced aerenchyma (*Symphiotrichum divaricatum*) was 302 cm^2^/g.

Our results for leaf δ^15^N suggest a trend of change over WIS categories in nitrogen sources for herbaceous species. δ^15^N tended to vary more over WIS categories for herbaceous species (*p *= .09) than woody species (*p *= .88). The δ^15^N of herbaceous species was greatest in the wettest WIS category (FACW) included in the analysis. This result suggests more variation in N sources over habitats for herbaceous species than woody species, perhaps due to shallower roots of herbaceous species. Frequency and magnitude of flooding and cycles of soil wetting and drying can alter riparian litter accumulation, nutrient dynamics, and discrimination against δ^15^N by soil microbes (Austin et al., 2004; Sponseller & Fisher, 2006). For example, Hall et al. (2015) reported δ^15^N enrichment in riparian species with submerged roots because of isotopic enrichment from benthic denitrification. Another factor possibly contributing to variability in N sources available to herbaceous species in our study is the density of woody species across the riparian landscape. Transitional riparian habitats along the Colorado River in Grand Canyon between the river edge and xeric uplands have greater woody vegetation and thus higher production of litter, likely contributing to faster biochemical processing and nutrient dynamics than more xeric habitats (Kennedy & Ralston, 2012; Sankey et al., 2015). The trend in δ^15^N for herbaceous species over WIS categories in our study suggests spatial variation in nitrogen sources that should be explored further.

Traits based on percent concentration of nitrogen and carbon in leaves and leaf C/N ratios did not differ among WIS categories or have interpretable trends with inundation or aridity and thus do not appear to be important riparian plant adaptations. Leaf nitrogen concentration was moderately high (>2%) for all species in all categories. Leaf carbon concentration ranged between 43% and 45% for species over all categories, except in the FACW category, where concentrations were about 40% for both herbaceous and woody species. This small difference in leaf nitrogen concentration resulted in little variation over WIS categories in C/N ratio, which is strongly associated with leaf decomposition rate (Pérez‐Harguindeguy et al., 2000; Taylor, Parkinson, & Parsons, 1989).

### 5.2 Multivariate traits

Principal components analysis based on traits of height, SLA, SSG, and seed mass showed moderate separation of species among WIS categories (Figure 4) and suggests a greater importance of SLA in distinguishing WIS categories than was indicated by the univariate analysis. The PERMANOVA distinguished hydrophytic species (OBL, FACW) from nonhydrophytic species (FAC, FACU, UPL) based on multivariate traits. Moderate correlation of SLA and SSG to PCO1 (*r* = −.66 and *r* = .42, respectively) and seed mass to PCO2 (*r* = −.35) suggest these traits explained substantial variation among categories. Specifically, hydrophytic species in Grand Canyon have a combination of lower SLA, SSG, and seed mass than nonhydrophytic species.

### 5.3 Trait correlations

SLA has been suggested as an easily measured surrogate of the leaf economics spectrum because it is correlated with other leaf traits, such as leaf nitrogen concentration, maximum photosynthetic rate, decomposition rate, and water use efficiency (Diefendorf et al., 2010; Pérez‐Harguindeguy et al., 2013; Reich, 2014; Wright et al., 2004). Our results are consistent with this suggestion for the 56 species with C3 photosynthesis we investigated. SLA was significantly correlated with δ^13^C, % leaf nitrogen, leaf C/N, and height. The weak but significant correlation (*r*_s_ = −.28, *p *= .003) between SLA and height was unexpected based on Westoby's leaf–height–seed scheme (1998), which postulates that these traits are orthogonal, or independent axes of trait variation. Significant correlation between SSG and percent leaf carbon (*r*_s_ = .51, *p *= .00005) suggests selection on stem and leaf traits collectively, consistent with the conclusions of other studies (Bucci et al., 2004; Ishida et al., 2008; Maherali, Moura, Caldeira, Willson, & Jackson, 2006). This collective selection is a likely driver for the positive correlation of percent leaf carbon and height (*r*_s_ = .34, *p *= .01), and the positive correlation of SSG and height (*r*_s_ = .20, *p *= .04) with greater height and canopy requiring greater support and overall carbon investment in tissue.

Our investigation of functional traits of vegetation that dominates riparian ecosystems along the Colorado River in Grand Canyon revealed five major findings. First, SSG, or what is also termed “stem density” or “wood density” (Chave et al., 2009), varies over WIS categories for both herbaceous and woody plants in a pattern consistent with an adaptive role of low‐density stem aerenchyma tissue in chronically inundated wetlands and dense cavitation‐resistant xylem in more arid habitats. This finding highlights SSG as an easily measurable, quantitative trait representing drought tolerance. Second, herbaceous and woody species adapted to arid habitats within riparian zone are shorter, which minimizes xylem tension in the canopy and thus mitigates against cavitation. Third, both woody and herbaceous species of arid habitats within the riparian zone have higher water use efficiency than species of wetter habitats. Fourth, OBL species have a distinct combination of multivariate traits (low SLA, low SSG, low seed mass) reflective of adaptation to frequent inundation. Lastly, our results provide quantitative support for classification of plant species into distinctly different hydrologic groups, such as OBL and obligate upland, or hydrophilic and nonhydrophilic, whereas classification into various FAC categories based on traits is more challenging due to overlap in traits.

## Author Contributions

TK, DM, BR, EP, and MM conceived the ideas and designed methodology; MM and EP led the data collection; MM, TK, and DM analyzed the data; TK and MM led the writing of the manuscript. All authors contributed critically to the drafts and gave final approval for publication.

## Data Accessibility

Trait data for the project have been archived at ScienceBase, https://doi.org/10.5066/F7BV7DTQ. ScienceBase is maintained by the United States Geological Survey.

## Conflict of Interest

Nothing to declare.
