# Supplementary material for: Variation in species‐level plant functional traits over wetland indicator status categories
Source: Ecol Evol. 2017 Apr 17;7(11):3732–44. doi: 10.1002/ece3.2975 (PMC5468150; doi:10.1002/ece3.2975)
Supplement: Supplementary file 1 [file ECE3-7-3732-s001.docx]

**Appendix S1 – Plant Species Collection Locations**

Species sampled from within the Grand Canyon were sampled during late September of 2014 and 2015. Species were collected outside of the Canyon and their collection dates are as follows: *Celtis laevigata* was collected along Red Tank Draw in the Verde Valley near Sedona, AZ on 2015-29-01. *Bromus tectorum* and *Taraxacum officinale* were collected on Northern Arizona University (NAU) campus on 2015-25-03 and 2015-03-06, respectively, in Flagstaff, AZ. *Scirpus americanus* was collected along Oak Creek near Sedona, AZ on 2015-21-05. *Equisetum arvense* and *Lactuca serriola* were collected from Sycamore Canyon, near Clarkdale, AZ on 2015-21-07. *Brickellia californica* and *Fallugia paradoxa* were collected near Leupp, AZ on 2015-04-06. *Elymus elymoides*, *Pascopyrum smithii*, *Hordeum jubatum*, and *Bromus arvensis* were collected near Prime Lake, near Flagstaff on 2015-26-06. *Typha domingensis* and *Distichlis spicata* were collected at the Sedona Wetlands Preserve on 2015-05-07, outside of Sedona, AZ. *Baccharis sarothroides* was collected along Red Rock Loop road in Sedona, AZ on 2015-05-07. *Mentha arvensis* was collected just south of Flagstaff at Marshall Lake, AZ on 2015-23-07. *Dasyochloa pulchella* was collected near Cottonwood, AZ on 2015-21-07. *Typha latifolia* was collected in Flagstaff, AZ on 2015-24-07. *Sporobolus airoides* and *Pleuraphis jamesii* were collected northeast of Flagstaff, AZ near Wupatki National Monument on 2015-15-07. *Agrostis exarata* was collected from Elden Spring in Flagstaff, AZ on 2015-15-07. *Verbena bracteata* was collected near Flagstaff airport on 2015-05-09.

**Appendix S2 – Latitude and Longitude of Collection Locations**

LAT and LON below are in decimal degrees in NAD83 projection. Points within Grand Canyon represent sandbar sites where collection was made. Collection locations made outside of Grand Canyon are approximate, as a gps was not used during these collections, but were estimated from Google Earth afterwards.

| **Species** | **LAT** | **LON** |
| --- | --- | --- |
| *Abronia elliptica* | 36.867580 | -111.589580 |
| *Acacia greggii* | 36.319931 | -111.860947 |
| *Achnatherum hymenoides* | 36.383915 | -111.858361 |
| *Agrosteris exarata* | 35.227830 | -111.601010 |
| *Alhagi maurorum* | 36.180292 | -111.814994 |
| *Allionia incarnata* | 36.867580 | -111.589580 |
| *Andopogon glomeratus* | 36.400845 | -112.552652 |
| *Apocynum cannabinum* | 36.383915 | -111.858361 |
| *Aremisia ludoviciana* | 36.594276 | -111.771605 |
| *Aristida arizonica* | 36.260500 | -111.826025 |
| *Artemisia dracunculus* | 36.469983 | -111.840030 |
| *Atriplex canescens* | 36.680847 | -111.739119 |
| *Baccharis emoryi* | 36.680847 | -111.739119 |
| *Baccharis salicifolia* | 36.365292 | -111.887839 |
| *Baccharis sarothroides* | 34.817450 | -111.846870 |
| *Baccharis sergiloides* | 36.390948 | -112.522193 |
| *Bebbia juncea* | 36.103538 | -111.829307 |
| *Bothriochloa barbinodis* | 36.530497 | -111.834483 |
| *Bouteloua barbata* | 36.319931 | -111.860947 |
| *Brickellia californica* | 35.300780 | -111.353010 |
| *Brickellia longifolia* | 36.680847 | -111.739119 |
| *Bromus arvensis* | 35.099020 | -111.528230 |
| *Bromus diandrus* | 36.867580 | -111.589580 |
| *Bromus rubens* | 36.867580 | -111.589580 |
| *Bromus tectorum* | 35.176400 | -111.656490 |
| *Celtis laevigata* | 34.680220 | -111.722630 |
| *Chloracantha spinosa* | 36.383915 | -111.858361 |
| *Conyza canadensis* | 36.530497 | -111.834483 |
| *Corispermum americanum* | 35.915422 | -113.334263 |
| *Cynodon dactylon* | 36.244262 | -112.507620 |
| *Dasyochloa pulchella* | 34.738930 | -111.984430 |
| *Datura wrightii* | 36.680847 | -111.739119 |
| *Dicoria canescens* | 36.383915 | -111.858361 |
| *Distichlis spicata* | 34.829490 | -111.897040 |
| *Eleocharis palustrus* | 34.959170 | -111.536380 |
| *Elymus canadensis* | 36.403684 | -111.879307 |
| *Elymus elymoides* | 35.099020 | -111.528230 |
| *Encelia farinosa* | 36.365292 | -111.887839 |
| *Enneapogon desvauxii* | 36.094961 | -111.840920 |
| *Ephedra torryana* | 36.680847 | -111.739119 |
| *Equisetum arvensis* | 34.832940 | -111.802020 |
| *Equisetum x ferrissii* | 36.383915 | -111.858361 |
| *Eragrostis curvula* | 36.319931 | -111.860947 |
| *Erodium cicutarium* | 36.867580 | -111.589580 |
| *Euthamia occidentalis* | 36.232017 | -112.977487 |
| *Fallugia paradoxa* | 35.300780 | -111.353010 |
| *Gutierrezia microcephala* | 36.383915 | -111.858361 |
| *Gutierrezia sarothrae* | 36.365292 | -111.887839 |
| *Hordeum jubatum* | 35.099020 | -111.528230 |
| *Hordeum murinum* | 36.867580 | -111.589580 |
| *Imperata brevifolia* | 36.179533 | -112.308696 |
| *Isocoma acradenia* | 36.260500 | -111.826025 |
| *Juncus arcticus* | 36.469983 | -111.840030 |
| *Juncus articulatus* | 36.403684 | -111.879307 |
| *Juncus torryi* | 36.365292 | -111.887839 |
| *Lactuca serriola* | 35.877530 | -112.067340 |
| *Larrea tridentata* | 36.232017 | -112.977487 |
| *Lepidium fremontii* | 36.867580 | -111.589580 |
| *Lepidium latifolium* | 36.365292 | -111.887839 |
| *Macharanthera canescens* | 36.338206 | -111.861984 |
| *Macharanthera pinnatifida* | 36.301843 | -112.786802 |
| *Melilotus albus* | 36.469983 | -111.840030 |
| *Mentha arvensis* | 35.099020 | -111.528230 |
| *Muhlenbergia asperfolia* | 36.365292 | -111.887839 |
| *Muhlenbergia microsperma* | 36.097718 | -112.182528 |
| *Muhlenbergia porterii* | 36.244508 | -112.517034 |
| *Nicotiana obtusifolia* | 36.867580 | -111.589580 |
| *Oenothera elata* | 36.530497 | -111.834483 |
| *Oenothera pallida* | 36.867580 | -111.589580 |
| *Panicum cappilare* | 35.166630 | -111.664130 |
| *Pascopyrum smithii* | 35.099020 | -111.528230 |
| *Phragmites australis* | 36.383915 | -111.858361 |
| *Piptatherum milaceum* | 36.088520 | -113.254341 |
| *Plantago lanceolata* | 36.867580 | -111.589580 |
| *Plantago major* | 36.867580 | -111.589580 |
| *Plantago patagonica* | 36.867580 | -111.589580 |
| *Pleuraphis jamesii* | 35.573870 | -111.487290 |
| *Pleuraphis rigida* | 36.244508 | -112.517034 |
| *Pluchea sericea* | 36.088520 | -113.254341 |
| *Polypogon monspeliensis* | 36.867580 | -111.589580 |
| *Polypogon viridis* | 36.469983 | -111.840030 |
| *Populus fremontii* | 36.143740 | -111.833960 |
| *Porophyllum gracile* | 36.244508 | -112.517034 |
| *Prosopis glandulosa* | 36.386708 | -111.8493294 |
| *Pseudognaphalium stramineum* | 36.469983 | -111.840030 |
| *Salex exigua* | 36.179533 | -112.308696 |
| *Salex goodinggii* | 36.094961 | -111.8409203 |
| *Salsola tragus* | 36.530497 | -111.834483 |
| *Sarcostemma cynanchoides* | 36.052215 | -113.349717 |
| *Schedonorous arundinaceus* | 36.403684 | -111.879307 |
| *Schismus arabicus* | 36.867580 | -111.589580 |
| *Scirpus americanus* | 34.832940 | -111.802020 |
| *Scirpus pungens* | 36.867580 | -111.589580 |
| *Sporobolus airoides* | 35.780890 | -111.425920 |
| *Sporobolus contractus* | 36.260500 | -111.826025 |
| *Sporobolus cryptandrus* | 36.594276 | -111.771605 |
| *Sporobolus giganteus* | 36.383915 | -111.858361 |
| *Sprobolus flexuosus* | 36.228074 | -112.507497 |
| *Stanleya pinnata* | 36.867580 | -111.589580 |
| *Stephanomeria pauciflora* | 36.594276 | -111.771605 |
| *Streptanthella longirostris* | 36.867580 | -111.589580 |
| *Symphiotrichum divaricatum* | 36.244508 | -112.517034 |
| *Tamarix ramosissima/chinensis* | 36.106679 | -112.147038 |
| *Taraxacum officinale* | 35.176400 | -111.656490 |
| *Thymophylla pentechaeta* | 36.867580 | -111.589580 |
| *Tridens muticus* | 36.106679 | -112.147038 |
| *Typha domingensis* | 34.877530 | -112.067340 |
| *Typha latifolia* | 35.203920 | -111.653040 |
| *Verbena bracteata* | 35.151420 | -111.660730 |
| *Xanthium strumarium* | 36.232017 | -112.977487 |

**Appendix S3 – Sample sizes**

For all following tables: SLA = specific leaf area, Height = average mature plant height, Seed Mass = average weight in grams per 1000 seeds, SSG = stem specific gravity, δ^13^C = ratio of [stable isotopes](https://en.wikipedia.org/wiki/Stable_isotope) [^13^C](https://en.wikipedia.org/wiki/Carbon-13) : [^12^C](https://en.wikipedia.org/wiki/Carbon-12), δ^15^N = ratio of [stable isotopes](https://en.wikipedia.org/wiki/Stable_isotope) [^15^N](https://en.wikipedia.org/wiki/Carbon-13) : [^14^N,](https://en.wikipedia.org/wiki/Carbon-12) C = percent carbon, N = percent nitrogen, C/N = carbon/nitrogen ratio. Row names represent wetland indicator status: OBL = Obligate, FACW = Facultative Wetland, FAC = Facultative, FACU = Facultative Upland, UPL = Upland.

Pooled samples

| Indicator | SLA | Height | Seed Mass | SSG | δ^13^C | δ^15^N | C | N | C/N |
| --- | --- | --- | --- | --- | --- | --- | --- | --- | --- |
| OBL | 7 | 7 | 6 | 7 | 2 | 2 | 2 | 2 | 2 |
| FACW | 17 | 17 | 16 | 17 | 13 | 13 | 13 | 13 | 13 |
| FAC | 20 | 20 | 18 | 20 | 10 | 10 | 10 | 10 | 10 |
| FACU | 23 | 22 | 22 | 23 | 10 | 10 | 10 | 10 | 10 |
| UPL | 43 | 43 | 39 | 40 | 21 | 21 | 21 | 21 | 21 |

Herbaceous species

| Indicator | SLA | Height | Seed Mass | SSG | δ^13^C | δ^15^N | C | N | C/N |
| --- | --- | --- | --- | --- | --- | --- | --- | --- | --- |
| OBL | 7 | 7 | 6 | 7 | N/A(2) | N/A(2) | N/A(2) | N/A(2) | N/A(2) |
| FACW | 12 | 12 | 11 | 12 | 8 | 8 | 8 | 8 | 8 |
| FAC | 17 | 17 | 15 | 17 | 7 | 7 | 7 | 7 | 7 |
| FACU | 18 | 17 | 17 | 18 | 6 | 6 | 6 | 6 | 6 |
| UPL | 31 | 31 | 28 | 28 | 15 | 15 | 15 | 15 | 15 |

Woody species

| Indicator | SLA | Height | Seed Mass | SSG | δ^13^C | δ^15^N | C | N | C/N |
| --- | --- | --- | --- | --- | --- | --- | --- | --- | --- |
| OBL |  |  |  |  |  |  |  |  |  |
| FACW | 5 | 5 | 5 | 5 | 5 | 5 | 5 | 5 | 5 |
| FAC | 3 | 3 | 3 | 3 | 3 | 3 | 3 | 3 | 3 |
| FACU | 5 | 5 | 5 | 5 | 4 | 4 | 4 | 4 | 4 |
| UPL | 12 | 12 | 11 | 12 | 6 | 6 | 6 | 6 | 6 |

NMS analysis

| Indicator | SLA | Height | Seed Mass | SSG |
| --- | --- | --- | --- | --- |
| OBL | 6 | 6 | 6 | 6 |
| FACW | 16 | 16 | 16 | 16 |
| FAC | 18 | 18 | 18 | 18 |
| FACU | 22 | 22 | 22 | 22 |
| UPL | 38 | 38 | 38 | 38 |
